# Frailty Syndrome and sarcopenia in older adults with and without type 2 diabetes mellitus in the municipality of Sinop, Mato Grosso: an epidemiological study *

**DOI:** 10.1590/1518-8345.6677.4077

**Published:** 2023-12-04

**Authors:** Alan Nogueira da Cunha, Maria Lucia Zanetti, Jair Licio Ferreira Santos, Rosalina Aparecida Partezani Rodrigues

**Affiliations:** 1 Universidade Federal de Mato Grosso, Instituto de Ciências da Saúde, Sinop, MT, Brasil.; 2 Universidade de São Paulo, Escola de Enfermagem de Ribeirão Preto, Centro Colaborador de la OPS/OMS para el Desarrollo de la Investigación en Enfermería, Ribeirão Preto, SP, Brasil.; 3 Universidade de São Paulo, Faculdade de Medicina de Ribeirão Preto, Ribeirão Preto, SP, Brasil.

**Keywords:** Aged, Diabetes *Mellitus*, *Mellitus*, Frailty, Sarcopenia, Primary Health Care, Nursing, Anciano, Diabetes Mellitus, Fragilidad, Sarcopenia, Atencion Primaria de Salud, Enfermería, Idoso, Diabetes *Mellitus*, *Mellitus*, Fragilidade, Sarcopenia, Atenção Primária à Saúde, Enfermagem

## Abstract

**Objective:**

to relate Frailty Syndrome and sarcopenia in older adults with and without type 2 diabetes mellitus and identify potential risk factors for frailty and sarcopenia.

**Method:**

this descriptive epidemiological study was conducted with 140 older adults in the municipality of Sinop, Mato Grosso, Brazil. The frailty phenotype was used for the assessment of Frailty Syndrome, and a physical assessment questionnaire with calf circumference measurement was used for the assessment of sarcopenia.

**Results:**

regarding Frailty Syndrome, a higher percentage was observed in older adults with type 2 diabetes mellitus compared to those without the disease (p = 0.00). Concerning the presence of sarcopenia, older adults with and without type 2 diabetes mellitus showed similar values, with no statistical significance (p = .74). Frailty Syndrome was associated with physical inactivity (95%CI: 3.29-56.55), age over 75 years (95%CI: 3.30- 27.82), low family income (95%CI: 1.80-50.98), and comorbidities (95%CI: 4.90-5.40). However, sarcopenia was associated with the presence of physical inactivity (95%CI: 1.26-10.44), low weight/ eutrophic (95%CI: 3.32-26.76), and malnutrition/nutritional risk (95%CI: 1.30-7.70) for older adults with and without type 2 diabetes mellitus.

**Conclusion:**

older adults with diabetes have a higher vulnerability to develop Frailty Syndrome, necessitating the adoption of preventive measures in primary healthcare.

## Abstract (es)

**Objetivo:**

relacionar el Síndrome de la Fragilidad y la sarcopenia en ancianos con y sin diabetes *mellitus* tipo 2; e identificar los potenciales factores de riesgo para la fragilidad y sarcopenia.

**Método:**

estudio epidemiológico descriptivo realizado en 140 ancianos del municipio de Sinop, Mato Grosso, Brasil. Para evaluar el Síndrome de la Fragilidad se utilizó el fenotipo de fragilidad y para la evaluación de la sarcopenia el cuestionario de evaluación física con medida de la circunferencia de la pantorrilla.

**Resultados:**

en lo que se refiere al Síndrome de la Fragilidad se obtuvo un mayor porcentaje para ancianos con diabetes *mellitus* tipo 2, cuando comparados con aquellos sin la enfermedad (p = 0,00). En relación a la presencia de sarcopenia, los ancianos con y sin diabetes *mellitus* tipo 2 presentaron valores semejantes, sin significación estadística (p = 0,74). El Síndrome de la Fragilidad presentó asociación con: inactividad física (IC95%: 3,29-56,55); intervalo etario superior a 75 años (IC95%: 3,30-27,82); baja renta familiar (IC95%: 1,80-50,98); y, comorbilidades (IC95%: 4,9-5,4). Entre tanto, la sarcopenia fue asociada a la presencia de la inactividad física (IC95%: 1,26-10,44), al bajo peso/eutrófico (IC95%: 3,32- 26,76) y a la desnutrición/riesgo nutricional (IC95%: 1,30-7,70), en los ancianos con y sin diabetes *mellitus* tipo 2.

**Conclusión:**

los ancianos diabéticos tienen mayor vulnerabilidad para desarrollar el Síndrome de Fragilidad, lo que requiere la adopción de medidas preventivas en la atención primaria a la salud.

## Abstract (pt)

**Objetivo:**

relacionar a Síndrome da Fragilidade e a sarcopenia em idosos com e sem diabetes *mellitus* tipo 2; e identificar os potenciais fatores de risco para fragilidade e sarcopenia.

**Método:**

estudo epidemiológico descritivo realizado com 140 idosos do município de Sinop, Mato Grosso, Brasil. Para a avaliação da Síndrome da Fragilidade, utilizou-se o fenótipo de fragilidade, e, para a avaliação da sarcopenia, o questionário de avaliação física com medida de circunferência da panturrilha.

**Resultados:**

quanto à Síndrome da Fragilidade, obteve-se maior percentual para idosos com diabetes *mellitus* tipo 2 quando comparado àqueles sem a doença (p = 0,00). Em relação à presença de sarcopenia, os idosos com e sem diabetes *mellitus* tipo 2 apresentaram valores semelhantes, sem significância estatística (p = 0,74). Síndrome da Fragilidade apresentou associação com inatividade física (IC95%: 3,29-56,55), faixa etária superior a 75 anos (IC95%: 3,30-27,82), baixa renda familiar (IC95%: 1,80-50,98) e comorbidades (IC95%: 4,9-5,4). Entretanto, a sarcopenia foi associada à presença da inatividade física (IC95%: 1,26-10,44), baixo peso/eutrófico (IC95%: 3,32-26,76) e desnutrição/risco nutricional (IC95%: 1,30-7,70) para os idosos com e sem diabetes *mellitus* tipo 2.

**Conclusão:**

os idosos diabéticos têm maior vulnerabilidade para desenvolver a Síndrome de Fragilidade, requerendo a adoção de medidas preventivas na atenção primária à saúde.

**Highlights:**

**(1)** Frailty and sarcopenia, important syndromes to be assessed in older adults.

**(2)** Older adults with T2DM have a higher vulnerability for the development of frailty.

**(3)** The nurse should implement measures to prevent frailty and sarcopenia.

## Introduction

The increase in the number of older adults worldwide is not considered a phenomenon exclusive to developed nations but also applies to countries in the process of development, such as Brazil ^(^ 1 ^)^. In the 1990s, life expectancy in Brazil was estimated to be 66 years, however, it could reach 78 years by 2030 ^(^ 2 ^)^. Estimates indicate that by the year 2050, the growth in the number of older adults will continue at a rate of 3.2% per year, making them 30% of the entire population ^(^ 2 ^)^. Recognizing demographic growth as a relevant and current process for society is essential to understand the needs presented by older adults, as the aging process involves changes in human body functions ^(^ 3 ^)^. 

This demographic transition that the country is undergoing contributes to the increased burden of non-communicable chronic health problems, including diabetes mellitus (DM), which stands out with high rates of comorbidities and mortality, particularly among older adults. Currently, there are 537 million adults worldwide (20 - 79 years of age), with 1 in 10 living with DM. It is estimated that by 2030, this number will increase to 643 million, and by 2045, it will reach 784 million ^(^ 4 ^)^. 

With the aging of the population, various syndromes are triggered, such as Frailty Syndrome and sarcopenia. Physical Frailty Syndrome is defined as a “clinical condition in which there is an increased vulnerability of an individual to the development of dependence and/or increased mortality when exposed to a stressor” ^(^ 5 ^)^. The presence of Frailty Syndrome in individuals over 60 years of age indicates the need for increased attention from healthcare providers due to the vulnerability of older adults and their predisposition to functional and physical deficits ^(^ 6 ^)^. One study showed a relationship between frailty, age, gender, level of education, marital status, economic status, the presence of heart disease, and hypertension, highlighting the need for early screening in primary healthcare ^(^ 7 ^)^. 

According to the European Working Group on Sarcopenia in Older People (EWGSOP), sarcopenia refers to low levels in the assessment of the parameters: muscle quantity and/or quality (measured by skeletal muscle mass), muscle strength (evaluated by handgrip strength), and physical performance (checked through the short physical performance battery or the individual’s gait speed) as an indicator of severity ^(^ 8 ^)^. 

A review study showed that in an aging society, the association between sarcopenia or frailty and type 2 diabetes mellitus (T2DM) is an important issue because the skeletal muscles of patients with the disease have a different distribution of myofibers compared to individuals without the disease. Its development is progressive and chronic, allowing for the implementation of effective care strategies to delay the condition ^(^ 9 ^)^. 

Therefore, older adults with T2DM may have a higher occurrence of Frailty Syndrome and sarcopenia, as these conditions are related to chronic diseases ^(^ 10 ^)^. The increase in older adults with sarcopenia in the diabetic population and its impact on quality of life affects psychosocial and physical health, making it a significant public health issue ^(^ 11 ^)^. International ^(^ 12 ^)^ and national ^(^ 7 ^,^ 13 ^-^ 14 ^)^ literature shows several studies on frailty and sarcopenia syndromes in older adults, although it is still insufficient for the diagnosis of DM. 

Accordingly, this study aimed to: relate Frailty Syndrome and sarcopenia in older adults with and without type 2 diabetes mellitus and to identify potential risk factors for frailty and sarcopenia.

## Method

### Study type

Descriptive epidemiological study, using the STROBE Statement (Strengthening the Reporting of Observational studies in Epidemiology) as a study reporting guide ^(^ 15 ^)^. 

### Location and population

The study was conducted with older adults registered and attended at the Jardim Botânico Primary Health Unit (PHU) in the municipality of Sinop, Mato Grosso (MT), Brazil.

### Period

Data collection was carried out from September 2019 to April 2020.

### Selection criteria

**Figure 1 -:**
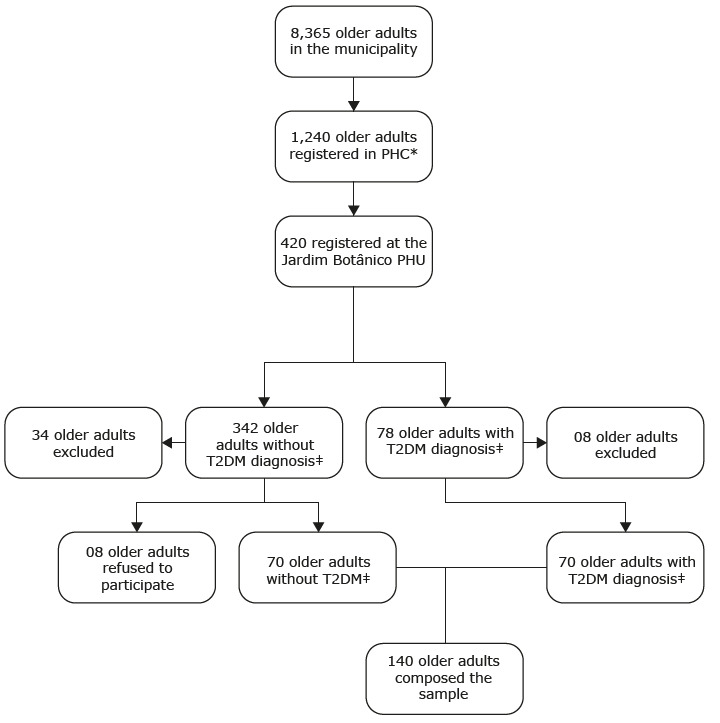
Sample selection criteria of the study

At the Primary Health Unit there were 420 older adults receiving care, of which 78 (18.6%) had a confirmed diagnosis of T2DM in their medical records. The selection of this PHU was due to the profile of the population attended, which was mostly composed of older adults. All older adults registered at the Jardim Botânico PHU, living at home, with a diagnosis of T2DM, and without the disease, who were able to communicate, were included. Older adults were excluded if they: had severe shortness of breath or other acute symptoms during the evaluation; were unable to move; had recent amputations and/or fractures (within the last three months); had severe sequelae from a stroke, with eight (8) individuals excluded, totaling 70 ( Figure 1). 

*PHC = Primary Health Center; †PHU = Primary Health Unit; ‡T2DM= Type 2 Diabetes mellitus

### Sample definition

For the sample definition, the total number of older adults with a diagnosis of T2DM was considered ( *n* = 78). Five of them were unable to communicate, two were bedridden, and one was wheelchair-bound, resulting in 70 older adults eligible for the study. One participant without DM was selected for each diabetic older adult, matched for sex (randomly chosen from medical records) and approximate age (with a three-year variation, either older or younger). Accordingly, 140 older adults composed the sample, with 70 participants having T2DM and 70 without the disease. Participants aged 60 years or over were considered older adults ^(^ 16 ^)^. 

### Study variables

The sociodemographic and clinical independent variables included: sex, age, self-reported skin color, marital status, years of education, living arrangements, family income, waist and hip circumference (cm), weight (kg), height (cm), BMI (body mass index), years of education, and HbA1C (glycated hemoglobin) (%), number of comorbidities, and nutritional status.

The dependent variables were: Frailty Syndrome (non-frail, pre-frail, and frail); and sarcopenia (with sarcopenia and without sarcopenia). Confounding variables included: age; BMI; and nutritional assessment. The independent variables were: inactivity; education level; and family income.

### Instruments used for data collection

To obtain sociodemographic and clinical data from the older adults, a questionnaire provided by the Research Center in Geriatrics and Gerontology of the University of São Paulo at Ribeirão Preto College of Nursing was used. This questionnaire included sociodemographic variables such as age, sex, marital status, living arrangements, education level, self-reported skin color, and income.

Frailty Syndrome was assessed using the frailty phenotype ^(^ 17 ^)^, which includes the criteria from the Cardiovascular Health Study: 1- Self-report of unintentional weight loss: This criterion was evaluated through the question: “In the past year, have you unintentionally (i.e., without diet or exercise) lost more than 4.5 kg?”; 2- Reduced physical strength: Evaluated by handgrip strength using a manual hydraulic dynamometer; 3- Self-report of exhaustion and/or fatigue: Two questions from the Brazilian version of the Center for Epidemiological Studies Depression Scale (CES-D) were used. 4- Gait speed: The test was administered to the older adult while seated in a 45 cm high chair, and they were instructed to stand up, walk a distance of 4.6 meters, return, and sit back down. 5- Decreased level of physical activity: Calculated based on the number of calories expended per week using the Minnesota Leisure Time Activity instrument, including activities like walking, household chores, and sports. Three levels of frailty were defined: frail if three (or more) criteria were met; pre-frail if 1 or 2 were met; and non-frail if there was no impairment ^(^ 17 ^)^. 

Sarcopenia was assessed using an instrument that measures muscle function and calf circumference called the SARC F+CC ^(^ 18 ^)^. It is defined by evaluations of muscle strength, history of falls, ability to rise from a bed or chair, ability to climb stairs, and calf circumference. The sum of the points characterizes the older adult as either having no signs suggestive of sarcopenia (0 to 10 points) or having signs suggestive of sarcopenia (11 to 20 points). 

Nutritional status was assessed using the Mini Nutritional Assessment (MNA) ^(^ 19 ^)^, which is divided into two parts: screening and global assessment. This assessment scores the older adult on three different nutritional indicators: 30.0 to 24.0, good nutritional status; 23.5 to 17.0, nutritional risk; and less than 17.0, malnourished ^(^ 19 ^)^. Glycated hemoglobin was assessed in the laboratory using the liquid chromatography method, considered the gold standard for this test. 

### Data collection

Throughout the data collection process, attention was given to quality control and standardization of the interviews and physical assessments. Continuous training of research assistants was conducted to ensure the internal validity of the data.

The team of interviewers consisted of one of the study authors and three students from the Federal University of Mato Grosso (UFTM), Sinop-MT Campus, enrolled in the Supervised Nursing Internship I course. During the training, each interviewer conducted a pilot assessment with five older adults randomly selected from the community (provided they met eligibility criteria) to address any questions or issues in the administration of questionnaires and procedures. These older adults were not part of the final sample.

Each older adult was personally invited to participate in the study, either in the waiting room of the PHC or through pre-scheduled phone contact. The selection of participants was performed randomly in the PHU reception waiting room as they sought healthcare, and by reviewing medical records to include 100% of older adults with a T2DM diagnosis. After interviewing the group of diabetic older adults, the search for participants without the disease was initiated in the medical records to match age and sex with the T2DM group.

### Data treatment and analysis

Data were analyzed using the STATA program version 14.0. Descriptive statistical analysis was applied to both the older adults with T2DM and those without the disease. Results were presented through absolute frequency ( *n*) and relative percentage (%) for the categorical variables, and mean, median, standard deviation, minimum, and maximum values for the continuous variables. Fisher’s exact test, and the Chi-square test were used to identify the relationship between variables. Bivariate regression was applied to select the independent variables, followed by multivariate logistic regression. The goodness-of-fit of logistic regressions was assessed using the Hosmer-Lemeshow test ^(^ 20 ^)^ with the option of five intervals for data grouping. A significance level of 95% confidence intervals and a significance coefficient of 5% ( *p* ≤ 0.05) were adopted, meaning that statistical significance was considered when α = 5%. 

### Ethical considerations

The project was reviewed and approved by the Research Ethics Committee (CEP) in accordance with Resolution 466/2012 of the National Health Council, under authorization number 3.279.884.

## Results

Regarding sociodemographic variables, among the 140 (100%) older adults with and without T2DM, the majority were female (55.7%), self-identified as having white skin color (57.1%), and were married (51.4%). In terms of age, there was a predominance of two age groups: 65 to 69 years (21.4%) and 80 years and over (21.4%). Most participants lived with a spouse (31.4%), had up to four years of education, and had a family income of three minimum wages (41.4%) ( Table 1). 

**Table 1 -:** Characterization of older adults with and without type 2 diabetes mellitus, according to sociodemographic variables (n = 140). Sinop, MT, Brazil, 2020

Variable		Older adults with T2DM*		Older adults without T2DM*	
		n	%	n	%
**Sex**	Male	31	44,28	31	44,28
	Female	39	55,71	39	55,71
**Age group (years)**	60 to 64	14	20,00	11	15,71
	65 to 69	15	21,40	17	24,28
	70 to 74	14	20,00	15	21,40
	75 to 79	12	17,14	10	14,28
	>80	15	21,40	17	24,28
**Self-reported skin color**	White	40	57,14	47	67,14
	Brown	24	34,29	21	30,00
	Black	4	5,71	5	7,14
	Indigenous	2	2,86	1	1,43
**Marital status**	Single	3	4,29	7	10.00
	Married	36	51,43	44	62,86
	Divorced	9	12,86	5	7,14
	Separated	2	2,86	1	1,43
	Widowed	20	28,57	13	18,57
**Education**	Up to 4 years of education	52	74,28	44	62,85
	More than 4 years of education	18	25,71	26	37,14
**Living arrangements**	Alone	15	21,43	6	8,57
	Only with spouse	22	31,43	30	42,86
	Spouse and child(ren)	11	15,71	9	12,86
	Spouse, children, son-in-law or daughter-in-law	2	2,86	5	7,14
	Only with child(ren)	8	11,43	3	4,29
	Three-generation arrangements	9	12.86	15	21,43
	Intra-generational arrangements	1	1,43	0	0
	Only with grandchildren	1	1,43	1	1,43
	Non-family members	1	1,43	1	1,43
**Family income in minimum** **wages ^†^ in Brazilian Reais**	1	13	18,57	8	11,43
	2	22	31,43	24	34,29
	3	29	41,43	30	42,86
	4	3	4,29	6	8,57
	5	2	2,86	2	2,86
	Do not know	1	1,43	0	0

*T2DM = Type 2 diabetes mellitus; †Actual minimum wage (in Brazilian Reais): R$ 1.040,00, Brazil, 2020

Regarding the clinical variables, the older adults with T2DM had higher mean values of waist circumference, hip circumference, weight, BMI, glycated hemoglobin, and the number of comorbidities when compared to those without the disease. However, the height was greater in older adults without the disease.

Analysis using the Chi-square test showed that BMI, classified as normal/overweight, had statistical significance ( *p* ≤ .00) with the presence of T2DM, as did HbA1C (greater than 6.5%) ( *p* ≤ .00), and the number of comorbidities (more than 5) ( *p* ≤ .00). The other variables did not show statistical significance ( Table 2). 

**Table 2 -:** Characterization of older adults with and without type 2 diabetes mellitus according to clinical variables (n = 140). Sinop, MT, Brazil, 2020

Older adults with T2DM* n = 70						Older adults without T2DM* n = 70				
**Variable**	Mean	Median	SD ^†^	Min ^‡^	Max ^§^	Mean	Median	SD ^†^	Min ^‡^	Max ^§^
**WC** ^||^	101.31	100.0	13.46	55.0	141.0	97.78	100.0	11.39	74.0	133.0
**HC** ^¶^	106.07	104.0	10.39	80.0	143.0	103.31	99.0	8.79	88.0	126.0
**Weight**	73.96	71.2	15.22	40.0	131.0	72.44	71.0	17.95	45.0	168.0
**Height**	160.95	164.0	12.37	120.0	186.0	164.04	163.0	9.01	143.0	181.0
BMI**	28.04	27.3	5.38	16.0	48.7	26.33	26.9	4.61	19.3	38.0
**HbA1c** ^††^	6.80	5.8	1.40	3.9	11.9	5.80	5.8	0.40	4.7	7.3
**Comorbidities**	7.00	5.0	3.70	0.0	16.0	5.00	5.0	2.70	0.0	16.0

*T2DM = Type 2 diabetes mellitus; †SD = Standard deviation; ‡Min = Minimum; §Max = Maximum; ||WC = Waist Circumference; ¶HC = Hip Circumference; **BMI = Body Mass Index; ††HbA1c = Glycated Hemoglobin

In the Frailty Syndrome analysis, a higher percentage was observed in the older adults with T2DM (47; 67.1%) compared to those without the disease (29; 41.4%), with statistical significance ( *p* = 0.00). Regarding the presence of sarcopenia, the older adults with and without the disease presented similar values (21; 30.0% with T2DM and 22; 31.4% without the disease), with no statistical significance ( *p* = 0.85) according to the Chi-square test. 

The sex variable did not show statistical significance considering the presence of frailty ( *p* = 0.28) and sarcopenia ( *p* = 0.57). However, the women presented a higher percentage of frailty (60; 42.2%) classified as pre-frail and frail, as well as a greater presence of sarcopenia compared to men. The 75 years and over age group showed statistical significance for both Frailty Syndrome ( *p* = 0.00) and sarcopenia ( *p* = 0.002). The majority of older adults in the 75 years and over age group, 78 (55.6%), were classified as pre-frail and frail. Regarding the presence of sarcopenia, the results were similar for both age groups. 

Regarding nutritional status, the majority of the older adults, 78 (55.6%), were classified as pre-frail and frail and presented a normal nutritional assessment. Therefore, there was no association with the presence of frailty ( *p* = 0.69). However, an association was found between nutritional assessment and malnutrition and sarcopenia ( *p* = 0.00). It is important to highlight that all of the individuals with comorbidities presented some degree of frailty ( *p* = 0.00). However, the majority of older adults with comorbidities did not present sarcopenia ( *p* = 0.32) ( Table 3). 

**Table 3 -:** Frailty and sarcopenia in older adults with and without T2DM*, by gender, age group, nutritional assessment, and comorbidities (n = 140). Sinop, MT, Brazil, 2020

Variables		Frailty *n*(%)				Sarcopenia *n*(%)		
		Not frail	Pre-frail	Frail	p	Without sarcopenia	With sarcopenia	p
**Sex**	Male	8 (5.7)	19 (13.5)	35 (25.0)	0.28	43 (30.7)	19 (13,5)	0.57
	Female	18 (12.8)	19 (13.0)	41 (29.2)		54 (38.5)	24 (17.1)	
**TOTAL n(%)**		26 (18,5)	38 (27.1)	76 (54.2)		97 (69.2)	43 (30.8)	
**Age group**	Up to 75	21 (15.0)	19 (13.5)	17 (12.1)	0.00	68 (48.5)	21 (15.0)	0.01
	75 and over	5 (3.5)	19 (13.5)	59 (42.1)		29 (20.7)	22 (15.7)	
**TOTAL n(%)**		26 (18.5)	38 (27.1)	76 (5402.0)		97 (69.3)	43 (30.7)	
**MNA** ^†^	Normal	21 (15.0)	27 (19.2)	51 (3604.0)	0.69 (*)	78 (55.7)	21 (15.0)	0.00 (*)
	Nutritional risk	5 (3.5)	10 (7.1)	22 (1507.0)		19 (13.5)	18 (12.8)	
	Malnourished	0(0.0)	1(0.7)	3(2.1)		0(0.0)	4(2.8)	
**TOTAL n(%)**		26(18.5)	38(27.1)	76(54.2)		97(69.3)	43(30.7)	
**Multimorbidities**	Up to 5	24(17.1)	30(21.4)	44(31.4)	0.00	65(46.4)	32(22.8)	0.32
	More than 5	2(1.4)	8(5.7)	32(21.4)		33(23.5)	10(7.1)	
**TOTAL n(%)**		26(18.5)	38(27.2)	76(52.8)		98(70.0)	42(30.0)	

*T2DM = Type 2 Diabetes Mellitus; †MNA = Mini Nutritional Assessment; *Chi-Square Test

Table 4 presents the multivariate logistic regression analysis of the frailty and sarcopenia assessment in older adults. The variables in the model were categorized as follows: BMI (underweight/normal weight and overweight); physical activity (physically active and sedentary); age group (up to 75 years and over 75 years); comorbidities (five or more morbidities); marital status (with a partner and without a partner); education (up to four years of education and more than four years of education); family income (up to 1 minimum wage and more than 1 minimum wage); nutritional assessment (normal nutritional status and nutritional risk/malnutrition). 

The association between frailty in the older adults with and without T2DM and the BMI, physical inactivity, age group, family income, comorbidities, and marital status variables are presented in Table 4. Multivariate regression analysis shows that the older adults with and without T2DM who were physically inactive had a thirteen times (OR = 13.64) higher likelihood of developing frailty ( *p* = 0.00). The likelihood of an older adult with or without diabetes mellitus developing frailty in the age group over 75 years and with higher family income was nine times higher. The likelihood of an older adult with five or more comorbidities developing frailty was four times higher (OR = 3.77). In the Hosmer-Lemeshow test for Frailty ( *N* = 140), with five groups, Hosmer-Lemeshow Chi ^2^ = 0.69; Prob > Chi ^2^ = 0.8747 (not significant). 

**Table 4 -:** Multivariate logistic regression of frailty and sarcopenia according to sociodemographic and clinical variables in older adults with and without T2DM* (n = 140). Sinop, MT, Brazil, 2020

Variable		*Odds Ratio*	*p* ^†^	95% IC ^‡^	
**Frailty**	Body Mass Index	0.18	0.00	0.06	0.54
	Physical inactivity	13.64	0.00	3.29	56.55
	Over 75 years age group	9.18	0.00	3.30	27.82
	Family income ^§^	9.58	0.00	1.80	50.98
	More than 5 comorbidities	3.77	0.01	1.36	10.48
	Marital status	1.29	0.59	0.49	0.54
**Sarcopenia**	Physical inactivity	3.64	0.01	1.26	10.44
	Over 75 years age group	1.45	0.71	0.51	4.10
	Body Mass Index	9.43	0.00	3.32	26.76
	Education less than 4 years	1.66	0.37	0.54	5.10
	Family income less than 1 MW ^§^	1.59	0.71	0.44	5.72
	Nutritional risk and/or malnourished	3.16	0.01	1.30	7.70

*T2DM = Type 2 Diabetes Mellitus; p† = Significance level; CI‡ = Confidence Interval; MW§ = minimum wage; Minimum wage (in Brazilian Reais): R$ 1,040.00, Brazil, 2020

In the multivariate regression, sarcopenia in the older adults with and without T2DM was associated with the physical inactivity, age group, BMI, education, family income, and inadequate nutritional status variables. Physical inactivity increased the risk of an older adult developing sarcopenia by three times (OR = 3.64) ( *p* = 0.01). On the other hand, the over 75 years age group showed a low relationship with the development of sarcopenia ( *p* = 0.71). Regarding the BMI of the older adults, being underweight and/or normal weight increased the odds of developing sarcopenia by nine times (OR = 9.43) compared to overweight individuals ( *p* = 0.00). Older adults with less than four years of education and family income below one minimum wage did not show statistical significance for the development of sarcopenia. Older adults at nutritional risk and/or malnourished had a three times higher likelihood (OR=3.16) of developing sarcopenia than those with adequate nutritional status. In the Hosmer-Lemeshow test for sarcopenia (n = 140), with 5 groups, Hosmer-Lemeshow Chi ^2^ = 0.61; Prob > Chi ^2^ = 0.8941 (not significant). 

## Discussion

This study investigated frailty syndrome and sarcopenia in older adults with and without T2DM and their potentially related factors in a sample of older adults living in the Central-West region of Brazil. Although sex did not show an association with the presence of frailty and sarcopenia, the women presented a higher likelihood of developing them. Similarly, advancing age and the presence of multimorbidity were associated with a higher likelihood of developing frailty. Another important factor to highlight is the nutritional risk/malnutrition assessed through the nutritional evaluation, which showed a significant relationship with sarcopenia. Additionally, frailty in the older adults was associated with sedentary behavior, the over 75 years age group, low family income, and multimorbidity. Sarcopenia, on the other hand, was associated with sedentary behavior, low weight/eutrophic status, and malnutrition/nutritional risk.

The older adults with T2DM presented higher values for waist circumference, hip circumference, weight, BMI (Body Mass Index), glycated hemoglobin (HbA1c), and the number of comorbidities when compared to those without the disease, with only frailty syndrome showing a relationship with the presence of T2DM.

When analyzing the sociodemographic conditions of the older adults with and without T2DM, it was observed that the majority were in the 60 to 79 years age group, consisting of white individuals, married, and with up to four years of education. There was a predominance of older adults with and without the disease living with a spouse and with a family income of three minimum wages. Regarding clinical variables, the older adults with T2DM showed higher mean values of waist and hip circumference, body weight, BMI, glycated hemoglobin (HbA1c), and the number of comorbidities when compared to those without the disease. These results are in line with the literature on DM ^(^ 21 ^)^. 

It is recognized that HbA1C is an important marker of glycemic control, requiring strict control, as altered values are associated with disease worsening and complications. In this study group, the mean value of HbA1C for the older adults with T2DM was 6.8%, and 5.6% for those without the disease. The study suggests that for healthy older adults, the desirable value is up to 7.5%, and for severely compromised older adults, it should be below 8.5%, which is higher than the limit for individuals without the disease, which is 5.6% ^(^ 22 ^)^. A value below 7% is considered adequate for individuals with T2DM, with complications tending to increase beyond this level. For older adults without the disease, the adequate value ranges from 4.0 to 5.6% ^(^ 22 ^)^. 

In the analysis using the Chi-Square test, older adults classified as eutrophic/overweight, HbA1C above 6.5, and having more than five comorbidities showed statistical significance for the presence of T2DM. The results of the sociodemographic and clinical variables can contribute to identifying the risk of developing frailty and sarcopenia in older adults with T2DM.

When analyzing Frailty Syndrome, a higher percentage was found in the older adults with T2DM compared to those without the disease. Regarding the presence of sarcopenia, the older adults with and without the disease showed similar values, with no statistical significance according to the Chi-Square test.

The sex variable did not show statistical significance with regard to frailty or sarcopenia; however, the women had a higher percentage of frailty, classified as pre-frail and frail, as well as the presence of sarcopenia compared to the men. When analyzing the presence of sarcopenia in the older women, the results were in line with systematic review and meta-analysis studies. These studies assessed the global prevalence of sarcopenia using both versions of the European Working Group on Sarcopenia in Older People, the EWGSOP and the EWGSOP2, and showed that the prevalence was higher in women when using the EWGSOP (17% vs. 12%). On the other hand, the prevalence of sarcopenia in men was higher when using the EWGSOP2 (11% vs. 2%). These studies indicate that the results obtained from the SARC-F+CC and EWGSOP2 instruments are similar ^(^ 23 ^)^. 

When analyzing the age group, it was found that older adults aged over 75 years showed statistical significance for both Frailty Syndrome and sarcopenia. It should be noted that the majority of older adults were classified as pre-frail and frail. Regarding the presence of sarcopenia, the results were similar in the two age groups investigated.

The relationship between frailty and age group is supported by the literature, and the results obtained corroborate those of other national and international studies. In one study conducted in Rio Grande do Sul, most older adults of higher age presented Frailty Syndrome, using the frailty phenotype criteria ^(^ 17 ^)^ ; however, in participants under 80 years of age, this condition was present in only half of the investigated sample ^(^ 24 ^)^. A longitudinal study conducted in Japan also showed that the incidence of frailty progressively increases with advancing age ^(^ 25 ^)^. 

Regarding nutritional status, it was found that most of the frail older adults had a normal nutritional status. This result differs from a study conducted in southern India, which showed that inadequate nutrient intake was associated with frailty ^(^ 26 ^)^. Most sarcopenic older adults had an adequate nutritional status assessed through the MNA (Mini Nutritional Assessment). However, a study conducted in Asia with people on hemodialysis found that inadequate nutrition was associated with the risk of osteoporosis and sarcopenia, suggesting the importance of a proper nutritional assessment and management to prevent bone and muscle-related complications ^(^ 27 ^)^. 

In the analysis of the comorbidities, it was found that the older adults with sarcopenia had a higher number of comorbidities compared to those without this condition. A longitudinal study conducted in the United Kingdom showed that comorbidities were associated with a higher risk of sarcopenia in 2,873 older adults over a 12-year follow-up period ^(^ 28 ^)^. The number of comorbidities in older adults can contribute to an increased degree of frailty. Accordingly, comorbidity and frailty appear to be similar and include a higher risk for healthcare utilization, disability, and mortality ^(^ 29 ^)^. 

Multivariate regression analysis showed that the older adults with and without T2DM who were inactive, in the age group over 75 years, with a family income below 1 MW, and with more than five comorbidities, had a higher chance of developing frailty.

Regarding physical inactivity and the presence of Frailty Syndrome, in accordance with the literature, an association between these two variables was found. Increased time doing physical activity provides a protective factor against frailty; however, there is a scarcity of clinical trial studies to confirm these results ^(^ 30 ^)^. An integrative review study that investigated the evidence for an association between frailty, physical activity, and exercise in older adults highlighted a relationship between these variables in the literature, as well as the potential for reversing frailty and improving other covariates with physical activity interventions ^(^ 31 ^)^. Likewise, it indicated an association between low levels of physical activity and frailty, decreased cognitive function, functional disability, mortality, and the number of comorbidities ^(^ 31 ^)^. 

Regarding family income, an association was found between low income and the occurrence of frailty. A study conducted in Pelotas, Rio Grande do Sul, Brazil, also found an association between frailty and low income ^(^ 32 ^)^. Similarly, another study also found frailty associated with sociodemographic conditions in older adults ^(^ 33 ^)^. It is evident that older individuals with low income face greater social vulnerability, difficulty accessing information, and challenges in accessing healthcare, nutrition, and leisure, factors that may explain the strong relationship between frailty and low income, as well as other factors associated with DM and other comorbidities. 

Regarding the relationship between the number of comorbidities and frailty, a longitudinal study conducted in a geriatrics outpatient clinic highlighted the prevalence of frailty and comorbidities as 66.2%, with a mean of 3.22 ± 1.78 simultaneous chronic morbidities. This investigation also showed that older adults with frailty and comorbidities had a lower survival rate ^(^ 34 ^)^. 

Another longitudinal study followed 6,425 older adults for 23 years and 6 months, concluding that 1,733 of them developed comorbidities, with 692 becoming frail and 611 developing some form of disability and passing away. The study found that comorbidities had a risk ratio of 2.38 for Frailty Syndrome ^(^ 35 ^)^. Comorbidities worsen the health of the person and generate difficulties in living their daily life independently ^(^ 36 ^-^ 37 ^)^. Therefore, it is recognized that managing older adults with type 2 diabetes mellitus is complicated due to comorbidities, reduced life expectancy, and exaggerated consequences of adverse effects of the treatment ^(^ 38 ^)^. 

Multivariate regression analysis showed that the older adults with and without T2DM who were physically inactive, underweight and/or eutrophic, at nutritional risk and/or malnourished had a higher chance of developing sarcopenia.

Regarding the assessment of physical activity level, evaluated through the Minnesota Leisure Time Activity, it was found that the older adults had a higher risk of developing sarcopenia. In line with our results, another study showed that older adults with a higher level of physical activity had a lower risk of sarcopenia (24.2%). This data may indicate that higher levels of physical activity constitute a protective factor against sarcopenia in older adults ^(^ 36 ^)^. An integrative review study highlighted that sedentary behavior is an important risk factor for the development of sarcopenia in older adults and emphasized the importance of physical activity as a preventive measure ^(^ 39 ^)^. It is well-known that the literature provides evidence of the benefits of physical activity for health ^(^ 40 ^-^ 41 ^)^. However, a combination of factors, including organic and nutritional factors, is necessary to achieve good results in the maintenance and strengthening of muscle in older adults. 

Another factor that may increase the occurrence of sarcopenia is advancing age. The results showed that the prevalence of sarcopenia according to age group was similar, with a slight increase in individuals above 75 years of age. A study conducted in Malaysia with 506 older adults with T2DM from primary healthcare showed that 28.5% of them presented sarcopenia. Among the associated factors were male gender and age ≥ 70 years, with ≥ 10 years of diabetes mellitus duration, low body mass index, involvement in light and moderate physical activities, and the use of fewer than five medications ^(^ 42 ^)^. 

The body mass index (BMI) is a universal indicator to assess whether an individual is at the ideal weight. However, this variable showed a strong association with sarcopenia, indicating that low weight and/or eutrophic older adults had a higher chance of developing sarcopenia. A study showed that individuals with low weight (BMI < 22) had a six-fold increase in the development of sarcopenia ^(^ 43 ^)^. Another study that evaluated the association between sarcopenia and risk factors, with 396,283 participants in the UK Biobank Baseline Clinic, showed that individuals below the ideal weight had a higher chance of having this condition ^(^ 44 ^)^. 

It is known that inadequate nutrition with insufficient nutritional and caloric quantities for muscle maintenance is a factor that contributes to the development of sarcopenia. Accordingly, the results of the present study show a strong relationship between older adults who presented malnutrition and/or nutritional risk and a higher chance of developing sarcopenia. Therefore, efforts should be made for older adults to consume an adequate portion of proteins for muscle maintenance as well as growth, which is beneficial for the metabolism ^(^ 45 ^)^. A review study highlighted the importance of physical activity in old age for the prevention and treatment of lean mass loss. This study showed that strength exercises in combination with protein supplementation for sarcopenic older adults provide various benefits and improve the quality of life, aiming for longevity and the prevention of malnutrition and other diseases ^(^ 46 ^)^. 

However, poor nutrition, with inadequate caloric and/or nutritional quantities, harms both muscle health, leading to sarcopenia ^(^ 47 ^)^, and the development of other morbidities, leading to body frailty ^(^ 48 ^)^, compromising the older adult’s immune system ^(^ 49 ^)^. It is recognized that the practice of physical activity and adherence to a healthy diet are non-pharmacological measures for the good control of T2DM and also assist in the prevention and control of Frailty Syndrome and sarcopenia. However, it is necessary to include and reinforce specific recommendations in the clinical practice of Gerontology and Geriatrics care, aiming to improve the quality of life of older adults and prevent these syndromes, and others ^(^ 50 ^)^. 

In summary, advanced age, low family income, and comorbidities are not changeable factors, however, are indicators that should be considered when planning care for preventing Frailty Syndrome and sarcopenia in older adults with and without T2DM.

The study’s limitations include: the cross-sectional study type, which did not allow causality to be established between variables; the sample size of older adults with and without DM, despite constituting the total registration in the PHU under study; the scarcity of literature on the relationship between Frailty Syndrome and sarcopenia in older adults with and without T2DM, and related factors that would allow for a more in-depth discussion of the results.

This study has the potential to contribute to developing innovative interdisciplinary approaches for ongoing care. It specifically emphasizes the importance of nurses in primary healthcare when consulting with older adults. Additionally, it highlights the significance of assessing Frailty Syndrome and sarcopenia in older adults with advanced age and T2DM as part of a comprehensive strategy for promoting health.

## Conclusion

It can be concluded that the older adults with type 2 diabetes mellitus were more vulnerable to the development of Frailty Syndrome, necessitating the adoption of preventive measures in primary healthcare. The results of this study have significant potential in primary healthcare, as they indicate the need for the implementation of interdisciplinary educational actions regarding Frailty Syndrome and sarcopenia. The nurse plays a pivotal role in health promotion, ensuring proper glycemic control, providing nutritional guidance, and recommending physical activities for older adults with T2DM. The inclusion of a systematic assessment of frailty and sarcopenia is recommended for older adults with and without T2DM of advanced age, as a tool used for promoting the health of the older adult.
